# Successful management of the hepatocellular carcinoma with inferior vena cava tumor thrombus: A case report

**DOI:** 10.1097/MD.0000000000026081

**Published:** 2021-05-28

**Authors:** Yuanjun Liu, Kunlin Xie, Jiaxin Li, Zhi Wang, Yong Zeng, Hong Wu

**Affiliations:** a Department of Liver Surgery & Liver Transplantation, State Key Laboratory of Biotherapy and Cancer Center, West China Hospital, Sichuan University and Collaborative Innovation Center of Biotherapy; b Department of Hepatobiliary Surgery, Suining Central Hospital, Suining; c Department of Clinical Research Management, West China Hospital, Sichuan University, Chengdu, Sichuan Province, China.

**Keywords:** case report, hepatocellular carcinoma, multidisciplinary team, stereotactic body radiotherapy

## Abstract

**Rationale:**

Hepatocellular carcinoma (HCC) with inferior vena cava tumor thrombus (IVCTT) is traditionally considered an advanced-stage cancer with a poor prognosis. There is no standard treatment for patients diagnosed as HCC with IVCTT.

**Patient concerns:**

A 52-year-old man was admitted to our hospital because of suspected hepatic mass during a health examination.

**Diagnoses:**

Computed tomography (CT) showed a hepatic mass approximately 4.3 cm × 6.3 cm in size located in segment VII of the liver, with thrombus in the inferior vena cava. The mass exhibited a pattern of early enhancement and washout on contrast-enhanced CT. Alpha-fetoprotein was 614.1 ng/mL (normal value, <8 ng/mL). The preoperative diagnosis was HCC with IVCTT.

**Interventions:**

Two months after stereotactic body radiotherapy combined with sorafenib therapy, a planned open anatomical resection of the right posterior lobe of the liver was performed.

**Outcomes:**

The patient is alive without disease 12 months after surgery, and the level of alpha-fetoprotein is normal.

**Lessons:**

The patient diagnosed as HCC with IVCTT was successfully treated by stereotactic body radiotherapy combined with molecularly targeted drugs followed by surgical treatment. If confirmed in future studies, this would suggest a promising strategy for the management of HCC with IVCTT.

## 1 Introduction

Hepatocellular carcinoma (HCC) is the third leading cause of cancer-related deaths worldwide, and its incidence is increasing in many countries.^[1–4]^ Patients with advanced HCC, especially those with inferior vena cava tumor thrombus (IVCTT), are considered to have an extremely poor prognosis, for which treatment with stereotactic body radiotherapy (SBRT) combined with molecularly targeted drugs has been proposed.^[5,6]^ Sorafenib, a tyrosine kinase inhibitor and a molecularly targeted drug, is generally recommended for patients with advanced HCC, with an expected median survival benefit of nearly three months.^[7,8]^ More effective local therapies are needed for patients with advanced HCC with IVCTT. Several studies have reported that SBRT achieved good results in the treatment of portal vein tumor thrombus^[9–11]^; therefore, we hypothesized that SBRT might also be effective for the control of IVCTT. Meanwhile, we report that HCC patients with IVCTT require surgical resection after SBRT combined with sorafenib.

## 2 Case presentation

**Figure 1:**
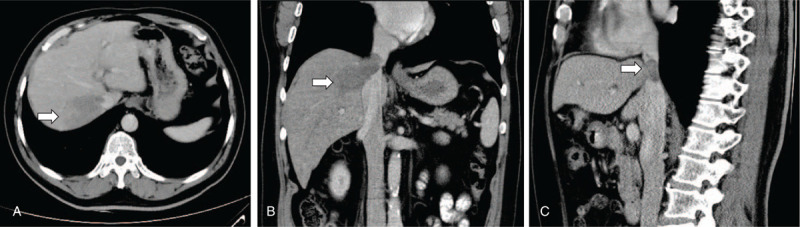
Dynamic contrast-enhanced CT scan findings. A: A hepatic mass approximately 4.3 cm × 6.3 cm in size in segment VII of the liver in the cross-section. B: A hepatic mass with thrombus in the IVC in the coronal plane. C: A thrombus in the IVC in the sagittal plane. IVC = inferior vena cava.

A 52-year-old man with hepatitis B virus (HBV) infection for more than 10 years visited a local hospital for a routine physical examination, and a hepatic mass was detected on abdominal ultrasound. He denied any fever, weight loss, or anorexia. A dynamic contrast-enhanced computed tomography (CT) scan revealed a hepatic mass approximately 4.3 cm × 6.3 cm in size located in segment VII of the liver, with thrombus in the inferior vena cava (IVC) (Fig. 1). Imaging also found evidence of portal hypertension, splenomegaly, and esophageal varices. The patient had a low HBV DNA copy number (<1.0 × 10^2^). His des-gamma-carboxy prothrombin or protein in vitamin K absence (PIVKA-II) level was 119 mAU/mL (normal range: 6.00–32.50 mAU/mL). Alpha-fetoprotein was elevated (614.1 ng/mL; normal value, <8 ng/mL), and tumor markers were negative for carcinoembryonic antigen and cancer antigen 19-9. Liver function was A5 according to the Child–Pugh classification. He regularly received entecavir antiviral therapy (0.5 mg orally every day) regularly for many years without a history of alcohol use. After evaluation by our multidisciplinary team, the patient was diagnosed with HCC with IVCTT, according to the Chinese standard (version 2017).^[12]^ Clinically, the patient had an Eastern Cooperative Oncology Group (ECOG) performance status score of 0, Chinese standard stage IIIa, Barcelona Clinic Liver Cancer (BCLC) stage C, and no ascites, encephalopathy, or other associated clinical symptoms. HCC with IVCTT is rare, and its prognosis is extremely poor. The multidisciplinary team recommended treatment with SBRT (radiation dose: 40 Gy/5 fractions over 5 days) combined with sorafenib therapy (400 mg twice daily) to shrink the HCC with IVCTT, preserve liver function, and prevent further deterioration.

**Figure 2:**
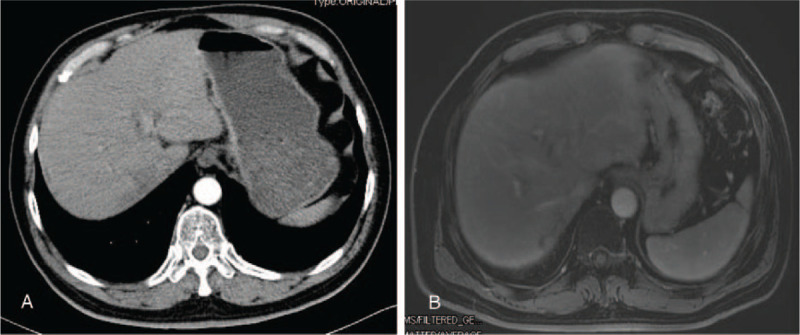
Dynamic contrast-enhanced CT (A) and MRI (B) scan findings after SBRT combined with sorafenib therapy (2 months later). The early phase of dynamic contrast-enhanced CT (A) and MRI (B) showed that the vascularity of the intrahepatic mass had been lost. No thrombus was found in the right hepatic vein or IVC. IVC = inferior vena cava, MRI = magnetic resonance imaging.

**Figure 3:**
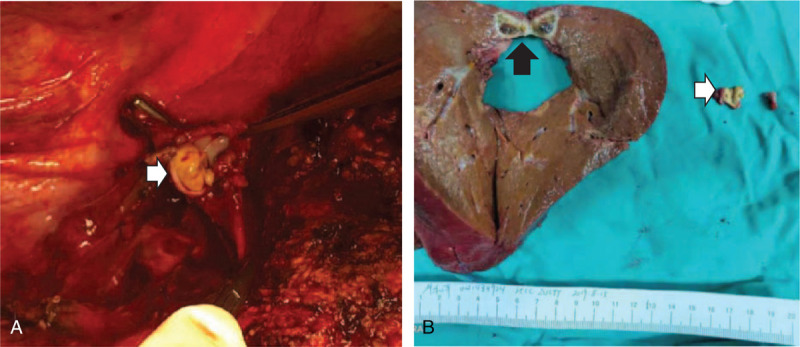
Intraoperative situation. A: A thrombus (white arrow) was found at the confluence of the right hepatic vein into the IVC after resection of the right posterior lobe of the liver. B: Postoperative specimens showed that the hepatic mass (black arrow, the size: approximately 1.4 cm × 1.3 cm × 0.6 cm) was located next to the right hepatic vein, and the thrombus (white arrow) was removed intact. IVC = inferior vena cava.

**Figure 4:**
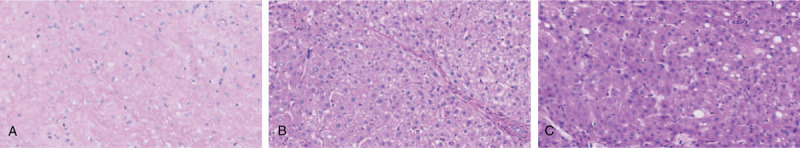
Histopathologic examination of the hepatic mass and liver tissue (hematoxylin and eosin staining, ×200). A: The image shows necrosis and fibrosis in the hepatic mass. B: Liver tissue within 1 cm of the edge of the hepatic mass. C: Liver tissue beyond 1 cm from the edge of the hepatic mass. Inflammatory cell infiltration dominated by foam cells, with hemosiderin deposition, which was consistent with the effects of radiotherapy, and no tumor residue was found.

**Figure 5:**
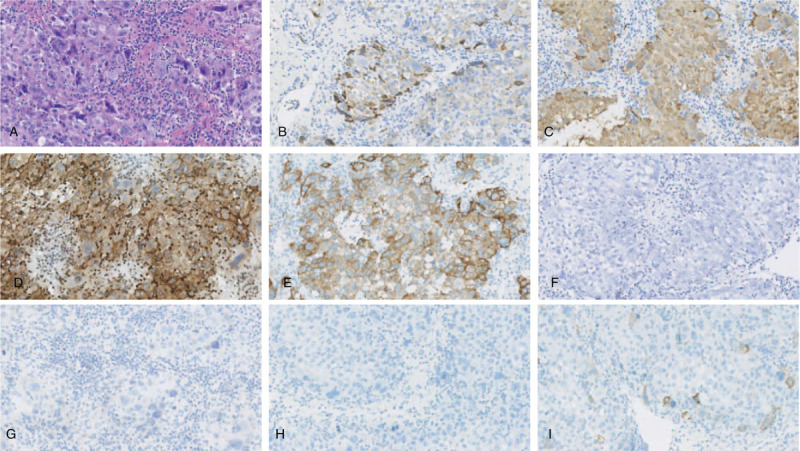
Histopathologic examination and immunohistochemical staining of tumor thrombus in the IVC. A: Cancer cells were found in the IVCTT (hematoxylin and eosin staining, ×200). B-I: The images (immunohistochemical staining, ×200) show Hepa (+), Arg (+), GPC-3 (+), CK8/18 (+), AFP (-), Muc-1 (−), CK7 (−), and CK20 (minority +), supporting HCC (poorly differentiated), respectively. AFP = alpha-fetoprotein, CK = cytokeratin, IVC = inferior vena cava, GPC = glypican.

The early phase of dynamic contrast-enhanced CT and magnetic resonance imaging, performed 2 months after treatment with SBRT combined with sorafenib therapy, showed that the vascularity of the intrahepatic mass had been lost. Moreover, no thrombus was found in the right hepatic vein or IVC (Fig. 2). Alpha-fetoprotein decreased to 57.74 ng/mL. After further consultation, the multidisciplinary team determined that, at that time, surgical resection would be the best option for the patient. The patient agreed to open anatomical resection of the right posterior lobe of the liver. During the operation, obvious liver cirrhosis was found, and the size of the mass was approximately 1.4 cm × 1.3 cm × 0.6 cm in size and was located next to the right hepatic vein. In addition, a thrombus was found at the confluence of the right hepatic vein into the IVC and was removed intact (Fig. 3). Histopathological examination (Fig. 4) revealed a free surgical margin, necrosis and fibrosis in the hepatic mass tissue, and inflammatory cell infiltration dominated by foam cells, with hemosiderin deposition, which was consistent with the reaction after radiotherapy. No tumor residue was found, and the surrounding liver showed nodular cirrhosis. Cancer cells were found in the IVCTT, and immunohistochemical staining (Fig. 5) showed Hepa (+), Arg (+), glypican-3 (+), cytokeratin (CK)8/18 (+), alpha-fetoprotein (-), Muc-1 (-), CK7 (-), and CK20 (minority +), supporting HCC (poorly differentiated). The patient recovered quickly without any complications and was discharged 1 week later. He received anti-HBV treatment and sorafenib therapy thereafter. The patient is alive without disease 12 months after the operation, with an ECOG performance status score of 1 and normal alpha-fetoprotein and PIVKA-II levels.

## 3 Discussion

Hepatic vein tumor thrombus is very common in the advanced stages of HCC and is an influencing factor of overall survival in patients. IVCTT is a further extension of the tumor thrombus in the main hepatic veins. In some cases, IVCTT may flow into the heart and lungs, leading to cardiac inflow tract obstruction, pulmonary embolism, and distant metastasis. Surgical treatment aims to achieve complete resection of the primary tumor and removal of the IVCTT with a sufficient functional liver remnant. However, studies of these procedures are limited because liver tumorectomy combined with IVC thrombectomy is challenging and associated with high morbidity and mortality. Advances in technology, diagnostics, pharmaceuticals, and surgery have changed the way cancer is managed. The complexity and number of treatments have increased to the point that no one specialty can provide complete patient care. The complexity of HCC should be managed in multidisciplinary settings according to guidelines established by the European Association for the Study of the Liver and the American Association for the Study of Liver Diseases.^[13,14]^ More than ever, high-quality management of cancer depends on patient access to timely evaluation and treatment and on multimodality therapy administered by a team of medical professionals.

In this case, the early phase of dynamic contrast-enhanced CT and magnetic resonance imaging, performed 2 months after treatment with SBRT combined with sorafenib therapy, showed that the vascularity of the intrahepatic mass had been lost. In addition, the tumor thrombus in the right hepatic vein and IVC was found to have shrunk. Appropriate specialists within the multidisciplinary team and timely therapeutic intervention are crucial for delivering optimal care to patients with this disease. The development of new staging criteria, such as the BCLC staging system and the Chinese standard (version 2017), has increased the effectiveness of treatment in patients with HCC. Systemic therapy, such as with the multi-targeted angiokinase inhibitor sorafenib, is the standard of care for patients with BCLC stage C HCC. Moreover, the 2010 European Society for Medical Oncology clinical practice guidelines recommend systemic therapy as the first-line treatment for patients with advanced and metastatic HCC (BCLC stage C).^[15]^

Although the modified Response Evaluation Criteria in Solid Tumors seems to be an effective tool for assessing the hemodynamics in typical HCCs after anti-angiogenic therapy, physicians should consider the possibility that the disappearance of intratumoral enhancement does not always indicate thorough necrosis, and living tissue could remain in a hypovascular intrahepatic mass.^[16]^ Another concern for physicians might be the possibility of conversion therapy after SBRT combined with sorafenib therapy. Considering its high objective response rate and low influence on liver dysfunction, SBRT combined with sorafenib therapy could achieve downstaging of tumors and lead to conversion therapy with surgical resection. When the main tumor is well controlled and the IVCTT has disappeared, additional surgical resection may achieve a clinical cure. The possibility of conversion surgery after SBRT combined with sorafenib therapy should be determined in real-world clinical practice. To achieve the best results from sorafenib, one must understand the radiological variations that occur during molecularly targeted therapy, to assess the precise treatment effects, and determine the optimal time to convert to a different treatment regimen. The combination of sorafenib and radiotherapy might produce better treatment outcomes in terms of tumor control. En bloc resection of the tumor with IVCTT can be achieved. Thus, compression and crushing of the IVCTT during the operation could be avoided, and new metastases caused by tumor thrombus to the lung and bone could be minimized. Multidisciplinary team management was associated with improved overall survival in patients with HCC, suggesting that such a team approach could improve patient outcomes.^[17]^

In this report, we describe the case of a patient diagnosed as HCC with IVCTT who underwent an open anatomical resection of the right posterior lobe of the liver combined with removal of the IVCTT after SBRT combined with sorafenib therapy. This suggests a promising treatment strategy for the management of HCC with IVCTT. However, additional studies, especially randomized controlled trials, are needed before they can enter routine clinical practice.

## Acknowledgments

The authors are most grateful for West China Biobanks, Department of Clinical Research Management, West China Hospital, Sichuan University for their support of human tissue samples.

## Author contributions

**Conceptualization:** Yong Zeng, Hong Wu.

**Data curation:** Zhi Wang.

**Investigation:** Kunlin Xie.

**Resources:** Zhi Wang.

**Supervision:** Yong Zeng.

**Validation:** Hong Wu.

**Visualization:** Hong Wu.

**Writing – original draft:** Yuanjun Liu.

**Writing – review & editing:** Yuanjun Liu, Kunlin Xie, Jiaxin Li, Zhi Wang, Yong Zeng, Hong Wu.
